# Viral Communities in the Global Deep Ocean Conveyor Belt Assessed by Targeted Viromics

**DOI:** 10.3389/fmicb.2019.01801

**Published:** 2019-08-21

**Authors:** Daniele De Corte, Joaquín Martínez Martínez, Mariana Silvia Cretoiu, Yoshihiro Takaki, Takuro Nunoura, Eva Sintes, Gerhard J. Herndl, Taichi Yokokawa

**Affiliations:** ^1^Research and Development Center for Marine Biosciences, Japan Agency for Marine-Earth Science and Technology (JAMSTEC), Yokosuka, Japan; ^2^Bigelow Laboratory for Ocean Sciences, Boothbay, ME, United States; ^3^Institute for Extra-cutting-edge Science and Technology Avant-garde Research (X-star), Japan Agency for Marine-Earth Science and Technology (JAMSTEC), Yokosuka, Japan; ^4^Research Center for Bioscience and Nanoscience (CeBN), Japan Agency for Marine-Earth Science and Technology (JAMSTEC), Yokosuka, Japan; ^5^Department of Limnology and Oceanography, Center of Functional Ecology, University of Vienna, Vienna, Austria; ^6^Instituto Español de Oceanografía, Centro Oceanográfico de Baleares, Palma, Spain; ^7^Department of Marine Microbiology and Biogeochemistry, Royal Netherlands Institute for Sea Research, Utrecht University, Utrecht, Netherlands

**Keywords:** targeted viromics, deep ocean circulation, viruses, deep ocean, next generation sequencing

## Abstract

Viruses are an abundant, diverse and dynamic component of marine and terrestrial ecosystems. In the ocean, viruses play a key role in the biogeochemical cycles and controlling microbial abundance, diversity and evolution. Recent metagenomics studies assessed the structure of the viral community in the upper ocean. However, little is known about the compositional changes in viral communities along the deep ocean conveyor belt. To assess potential changes in the viral community in the global deep-water circulation system, water samples were collected in the core of the North Atlantic Deep Water (NADW) (∼2,500 m) and Pacific Antarctic Bottom Water (∼4,000 m). Microbial and viral abundance were evaluated by flow cytometry. Subsequently, flow cytometry was used to sort virus-like particles and next generation sequencing was applied to build DNA libraries from the sorted virus populations. The viral communities were highly diverse across different oceanic regions with high dissimilarity between samples. Only 18% of the viral protein clusters were shared between the NADW and the Pacific Antarctic Bottom Water. Few viral groups, mainly associated with uncultured environmental and uncultured Mediterranean viruses were ubiquitously distributed along the global deep-water circulation system. Thus, our results point to a few groups of widely distributed abundant viruses in addition to the presence of rare and diverse types of viruses at a local scale.

## Introduction

Viruses shape marine ecosystems by controlling their host abundance and diversity through cell lysis and generate and maintain diversity through horizontal gene transfer (Angly et al., 2006; Rohwer and Thurber, 2009; Zeng and Chisholm, 2012). Moreover, viral lysis influences oceanic productivity by promoting organic matter and nutrient cycling through the release of intracellular material from the host cells (Middelboe et al., 1996; Middelboe and Lyck, 2002).

Several parameters affect the distribution of viruses in the marine environment (De Corte et al., 2012, 2016; Brum et al., 2015). The presence of a suitable host is key to perpetuate the viral “life cycle.” However, other variables, such as temperature (Parada et al., 2007), UV radiation (Wommack et al., 1996) and salinity (Kukkaro and Bamford, 2009), influence the time span that viruses remain active in the ambient water and capable of infecting a host. The effects of these biotic and abiotic factors have been studied in surface waters (Bongiorni et al., 2005; De Corte et al., 2011) and, infrequently, in the deep ocean, where low temperature leads to relatively slower virus decay rates than in surface waters (Parada et al., 2007). The longer bacterial generation time and slower viral decay rates in the deep ocean (Parada et al., 2007; De Corte et al., 2012), together with the limited vertical mixing of the deep-water masses, suggest that oceanic currents and the thermohaline circulation may play an important role in influencing the global distribution of viruses and their hosts (McGillicuddy et al., 2007; Sintes et al., 2013, 2015; Sul et al., 2013; Brum et al., 2015; Frank et al., 2016).

**FIGURE 1:**
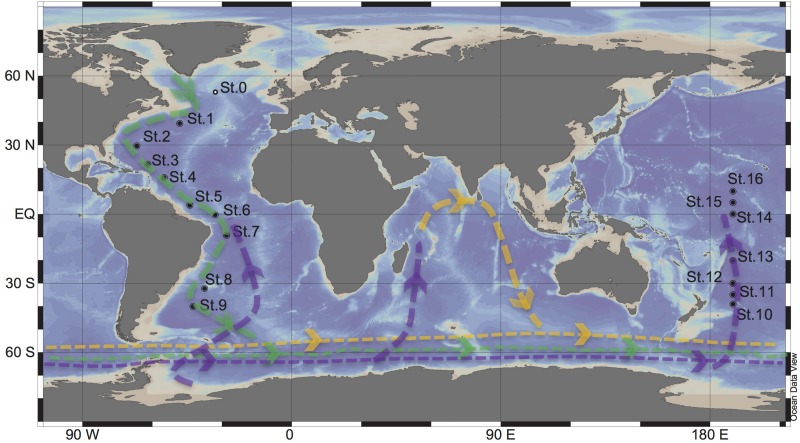
Sampling stations for targeted viromic analysis occupied during the GEOTRACES research expeditions 1, 2 and 3 (2010–2011) in the Atlantic (St 1 to 9) and during KH13-7 and the KH14-3 cruises (2013–2014) in the Pacific (St 10 to 16). The lines represent the simplified scheme of the deep thermohaline circulation, modified from Talley (2013): Green = North Atlantic Deep Water (NADW), purple = Antarctic Bottom Water (AABW), Yellow = Indian Deep Water (IDW). St0 sampled during the MEDEA2 cruise and used for standard viromic analysis is also shown.

The global overturning circulation (Figure 1) is a system of oceanic currents driven by wind, density and mixing processes (Schmittner et al., 2007; Talley, 2013). The North Atlantic Deep Water (NADW) is formed in the North Atlantic Ocean through the sinking of cold and dense waters, and moves southward toward the Southern Ocean (Talley, 2013). The second limb of the deep overturning circulation system is the Antarctic Bottom Water (AABW). The AABW is formed in the Southern Ocean via mixing of NADW, Indian Deep Water, Pacific Deep Water, and shelf waters originating from Antarctic shelf systems (Talley, 2013).

Viruses, as part of the dissolved organic matter pool (DOM; operationally defined as the organic matter that passes through a 0.2 μm filter), should behave conservatively and reflect the different formation sites of the deep waters (Bercovici and Hansell, 2016). However, it is important to note that certain local effects, such as particle export and *in situ* production (Nagata et al., 2000; Hansell and Ducklow, 2003; Yokokawa et al., 2013), play an important role in determining bacterial and viral abundance and community composition. Sinking particles provide a source of fresh substrate for microbes in the deep sea (Cho and Azam, 1988; Karl et al., 1988; Follett et al., 2014). Hence, this organic matter might be especially important in the Pacific Deep Water of the North Pacific with its low DOM concentration (Hansell, 2013).

DNA viruses lack conserved marker genes, hindering the assessment of their biogeographical patterns. The dispersal of viruses across the oceans has only been recently explored (Angly et al., 2006; Hurwitz and Sullivan, 2013; Martinez-Hernandez et al., 2017; Gregory et al., 2019). Moreover, most studies addressing viral community composition are limited to the upper ocean layers (Angly et al., 2006; Brum et al., 2015; Hurwitz et al., 2015). Due to methodological limitations, virus-host interactions in the dark ocean have mainly been assessed by microbial and viral enumeration (De Corte et al., 2012; Wigington et al., 2016; Lara et al., 2017). Therefore, little is known about the changes of viral communities along the deep ocean conveyor belt circulation system.

Although viromics (metagenomics of viral communities) is increasingly used to study viral diversity and function in the marine ecosystems (Breitbart et al., 2004; Breitbart and Rohwer, 2005; Hurwitz and Sullivan, 2013; Brum et al., 2015), it has its limitations. Viromics typically requires a large sample volume, which may limit the number of samples that can be collected and processed in a timely manner. Moreover, filtration of water samples to physically separate the viral fraction from other microorganisms may lead to the loss of a fraction of the viral community, in particular of giant viruses, while some small bacteria might pass through the filter (Martinez Martinez et al., 2014).

The aim of this study was to assess the composition of the viral communities along the deep-water masses of the thermohaline circulation using a targeted viromics approach. Seawater samples from the core of the NADW (∼2500 m) and the Pacific AABW (∼4000 m) were used to discriminate and sort virus-like particles (VLPs) by flow cytometry. The sorted particles were subsequently used to produce DNA libraries for next-generation sequencing (Martinez Martinez et al., 2014). Our analyses provide insight into the composition of viral communities through the circulation path of two major deep-water masses flowing from the Atlantic Ocean through the Southern Ocean to the Pacific. Our data suggest that the thermohaline circulation together with the flux of sinking particles from the epipelagic layer shape the viral community of the deep ocean.

## Materials and Methods

### Study Area and Sampling

North Atlantic Deep Water (∼2500 m) samples were collected at nine stations during the GEOTRACES cruises 1–3 (2010–2011) in the Atlantic Ocean, on board of the R/V *Pelagia* and R/V *James Cook.* Antarctic Bottom Water (∼4000) samples were collected at seven stations during the KH13-7 and KH14-3 (2013–2014) cruises in the Pacific Ocean on board of the R/V HAKUHO MARU (Figure 1). Sampling was performed using a rosette water sampler equipped with 12 or 24 Niskin bottles with the capacity of either 12 or 25-L, a CTD system (conductivity-temperature-depth; SBE43 Seabird, Bellevue, WA, United States), and a dissolved oxygen sensor.

Two sets of 2-mL water samples from each depth and station were fixed with 0.5% (Atlantic samples) and 1% glutaraldehyde (Pacific samples) held at 4°C for 30 min and flash-frozen in liquid nitrogen thereafter. The samples were stored at −80°C until further processing.

### Determination of Microbial Abundance and Sorting of Viruses

Flow cytometry following nucleic acid staining (SYBR Green I) was used to enumerate both, viruses and microbes as described elsewhere (Del Giorgio et al., 1996; Marie et al., 1999; Brussaard, 2004). According to their respective signature in the cytogram of green fluorescence *vs*. side scatter, two different viral clusters (high and medium fluorescence VLPs) were discriminated (Supplementary Figure S1).

A second set of glutaraldehyde-fixed samples was used for sorting VLPs. After thawing on ice, the samples were rinsed three times with 1xTE Low-EDTA buffer (10 mM Tris–HCl, 0.1 mM EDTA, pH 8.0) using Amicon 3 kDa centrifuge filters (Millipore) to remove excess glutaraldehyde, which may prevent or decrease the efficiency of downstream DNA amplification (Das et al., 2014). Sorting of viruses was conducted at the JJ MacIsaac Facility for Aquatic Cytometry of the Bigelow Laboratory for Ocean Sciences, with an Influx flow cytometer (BD Biosciences) following an established protocol (Martinez Martinez et al., 2014). Briefly, SYBR Green I stained viruses were discriminated by triggering on side scatter and sorted according to their signature in green fluorescence and side scatter signals. The gain on the 531 nm (green) emission photomultiplier (PMT) was 42.21 and the threshold was set at 0.18. A blank to determine the background noise level was run prior to the samples, and the sorting gates were determined to ensure particle sorting above the background signal region (Supplementary Figure S1). The “single 1 drop” mode was used to avoid non-target particles in the target particle droplet and the adjacent droplets. Approximately 6,000 to 10,000 virus particles from each cluster were sorted into 1.5 mL LoBind, sterile microcentrifuge tubes (Eppendorf) for viromics analysis.

### Whole Genome Amplification of Sorted Viral DNA and Next-Generation Sequencing (Sorted Viromes)

Aliquots containing between ∼1,000 and ∼5,000 sorted virus particles (detailed information in Supplementary Table S1) from each viral cluster (high and medium nucleic acid fluorescence virus particles) were drawn from each microcentrifuge tube and subjected to whole genome amplification using Illustra Single Cell GenomiPhi DNA Amplification kit (GE Life Sciences) following the manufacturer’s protocol. Subsequently, the amplified genetic material was purified with a PCR purification kit (Qiagen), quality checked by 2% agarose gel electrophoresis and quantified using a Qubit fluorometer (ThermoFisher). The DNA libraries were constructed using KAPA hyper DNA Library Preparation Kit (with thermocycling consisting of 12 cycles) following the manufacturer’s recommendations and sequenced using Illumina MiSeq high throughput sequencing (2 × 250 bp paired-end platform).

### Standard Viromes

Seawater samples were collected at Station 0 in the North Atlantic at 25 m (220 L), the oxygen minimum zone (OMZ [460 m], 260 L) and 2,000 m (460 L) during the MEDEA-2 cruise in July 2012 (Figure 1). The seawater samples were serially pre-filtered through 1.0 and 0.2 μm polycarbonate filters (Millipore) to remove most microorganisms and to enrich the viral fraction. Subsequently, the <0.2 μm filtrate, containing the viral particles, was concentrated by tangential flow filtration using a 100 kDa cutoff cartridge (Millipore) to a final volume of ∼150 mL. The concentrates were stored at −80°C until further processing. Nucleic acids from the concentrates were extracted and purified using the phenol/chloroform protocol. The quality and quantity of the extracted DNA was checked by a Nanodrop spectrophotometer (ThermoFisher Scientific). Standard virome DNA libraries were prepared with NEBNext DNA Library Prep Master Mix with thermocycling consisting of 12 cycles, and sequenced at Eurofins Genomics using an Illumina HiSeq high throughput sequencer (2 × 100 bp paired-end platform).

### Bioinformatic Analyses

The obtained reads were quality trimmed and controlled using trimmomatic (Bolger et al., 2014) with Phred33. Reads shorter than 36 bp were removed from the analysis. The reads from the sorted viromes were first digitally normalized using bbmap^1^, and subsequently taxonomically classified with the Kaiju classifier (Menzel et al., 2016) using the non-redundant NCBI database that included viruses, prokaryotes, fungi and microbial eukaryotes.

Assembly of the filtered reads was performed using metaSpades (Nurk et al., 2017) with 21, 33, 55 k-mers size. The obtained contigs were mined for viral signal by VirSorter (Roux et al., 2015) implemented in iVirus^2^ (Bolduc et al., 2017) against their virome database (Roux et al., 2015) and Viral Single Amplified Genomes (vSAGs) obtained from Martinez-Hernandez et al. (2017). Only phage contigs (from category 1 to 3) larger than 1,500 bp were retained for further analysis. The viral contigs are publicly available in the iMicrobe platform^3^. To create a read count per contig table, the reads were mapped back to the obtained viral contigs using Bowtie 2 (local alignment, sensitive setting) (Langmead and Salzberg, 2012) implemented in iVirus (Bolduc et al., 2017). Anvio’o v.5.2 (Eren et al., 2015) was used to generate coverage profiles of the mapped reads into viral contigs using only the second and third quartile coverage value from each contig. Open reading frames (ORFs) of the viral contigs were identified using Prodigal (Hyatt et al., 2010). The ORFs taxonomic affiliation was determined with BLASTx (Altschul et al., 1997) with an *e*-value cut-off of <10^–5^ using Uniref 100 viral database. Contigs affiliated with the Taterapox virus and Propionibacterium phages were discarded from the analysis as potential laboratory contaminants (Woyke et al., 2011; Martinez Martinez et al., 2014). Additionally, the obtained contigs were classified by Genome-BLAST Distance Phylogeny (GBDP) (Meier-Kolthoff et al., 2013) using the settings recommended for prokaryotic viruses (Meier-Kolthoff and Goker, 2017). The evolution trees obtained from the intergenomic distances were calculated on FASTME (Lefort et al., 2015) with branch support inferred from 100 pseudo-bootstraps. The obtained trees were rooted at the midpoint (Farris, 1972) and visualized with FigTree (Rambaut, 2006). Taxon classification was estimated with the OPTSIL program (Goker et al., 2009) with the recommended clustering thresholds (Meier-Kolthoff and Goker, 2017) and an *F* value of 0.5 (Meier-Kolthoff et al., 2014).

The sequence data generated are publicly available in the DDBJ database under the accession number DRA008218.

### Viral Recruitment Analysis

The sorted and standard viromes were compared to five viral genomes and four environmental samples (three vSAGs and one assembled genome) isolated from marine ecosystems. From each virome, 1.5 million randomly subsampled reads were recruited against reference sequences using tBLASTx with an *e*-value cut-off of <10^–5^. Both the average and percentage coverage values were calculated from the nucleotide alignment positions of the reads (obtained from the tBLASTx results) against the reference genome.

### Protein Clustering Analysis

The viral contigs were quality controlled using QUAST, allowing one ambiguity and the threshold for misassembles set to 1000 bp (Gurevich et al., 2013). The ORFs were predicted and annotated using PROKKA (Seemann, 2014) optimized in house for virus detection. Briefly, PROKKA was supplemented with a prodigal (Hyatt et al., 2010) in house-trained HMM model of the non-redundant Reference Viral Database (RVDB version April 2018, Goodacre et al., 2018) containing viral, virus-like and virus-related sequences. The predicted ORFs were clustered to existing proteins obtained from the Reference Viral (Goodacre et al., 2018) and Tara Ocean Database (Brum et al., 2015) using CD-HIT-2D (Li and Godzik, 2006) with a sequence cutoff of 60% and a coverage cutoff of 80%. To create a table with the read counts per protein clusters, the reads were mapped back to the obtained ORFs using Bowtie 2 (Langmead and Salzberg, 2012).

### Statistical Analysis

Analysis of variance (ANOVA test) was performed to test possible variations between each water mass. The richness of the viral contigs and viral protein clusters were calculated from the coverage table using “Vegan” package (Oksanen et al., 2018) implemented in R software v.3.4. Jaccard Dissimilarity index was calculated from the subsampled (200,000 reads per sample) coverage table (obtained from the viral contigs and protein clusters) using the “Vegan” package.

The presence of specific protein clusters in the different samples was analyzed with the “Venn” package in the R software using presence/absence data.

## Results

### Environmental and Microbial Parameters

The NADW and the Pacific Antarctic Bottom Water (PAABW) water differed in their physico-chemical characteristics. NADW exhibited higher temperature, salinity and dissolved oxygen concentrations (2.9 ± 0.5°C, 34.9 ± 0.1, 233 ± 13 μmol/kg, respectively) than the PAABW (1.3 ± 0.13°C, 34.7 ± 0.0, 172 ± 23 μmol/kg, respectively) (Table 1). Prokaryotic and viral abundance were significantly higher in the NADW (1.4 ± 0.9 × 10^4^ mL^–1^, 5.2 ± 2.0 × 10^5^ mL^–1^, respectively) than in the PAABW, with prokaryotic and viral abundance of 0.7 ± 0.1 × 10^4^ mL^–1^ and 1.2 ± 0.2 10^5^ mL^–1^, respectively (*T*-test, *p* < 0.05). The viral to prokaryotic ratio (VPR) was also significantly higher in the NADW (45 ± 18) than in the PAABW (16 ± 2) (*T*-test, *p* < 0.05).

**TABLE 1:** Physico-chemical and biological characteristics of the North Atlantic Deep Water (NADW) and Pacific Antarctic Bottom Water (PAABW) sampled in the Atlantic and Pacific Ocean, respectively.

			**Environmental Parameters**					**Microbial Counts**		
	**Samples**	**N**	**Water mass**	**Depth (m)**	**Temperature (°C)**	**Salinity**	**Oxygen (μmol/kg)**	**Prokaryotic Abundance (×10^4^ mL^−1^)**	**Viral Abundance (×10^5^ mL^−1^)**	**VPR**
					**Average** ±**SD**	**Average** ±**SD**	**Average** ±**SD**	**Average** ±**SD**	**Average** ±**SD**	**Average** ±**SD**
Atlantic	North Atlantic	4	NADW	2500	3.1 ± 0.2	34.9 ± 0.0	234 ± 3.4	0.8 ± 0.2	4.8 ± 1.1	61 ± 5
	Equator	2	NADW	∼2500	2.9 ± 0.1	34.9 ± 0.0	240 ± 13.2	1.5 ± 0.8	3.6 ± 1.3	29 ± 23
	South Atlantic	3	NADW	2500	2.5 ± 0.7	34.9 ± 0.1	227 ± 23	2.2 ± 1.2	7.0 ± 2.3	34 ± 7
Pacific	South Pacific	4	AABW	∼4000	1.2 ± 0.1	34.7 ± 0.0	191 ± 3.6	0.8 ± 0.1	1.3 ± 0.2	17 ± 2
	Equator	2	AABW	∼4000	1.4 ± 0.0	34.7 ± 0.0	146 ± 3.0	0.8 ± 0.2	1.2 ± 0.1	17 ± 1
	North Pacific^∗^	1	AABW	∼4000	1.4	34.7	150	0.5	0.8	15

*^∗^A single data point was collected at this station. Therefore, average and standard deviation are not reported. Average ± standard deviation (SD) and number of samples (N) are indicated.*

### Taxonomic Composition of the Sorted Viromes

Unclassified reads accounted for 52 ± 22% of the total sequences, followed by Bacteria (40 ± 23%), Eukarya (4 ± 2%), viruses (3 ± 2%) and Archaea (0.6 ± 0.2%). Consequently, excluding the reads with no or unknown homology, viruses accounted for ∼7% of the DNA library. The North Atlantic and North Pacific libraries comprised the lower proportion of viral reads with 2% and 1% of their corresponding libraries, respectively. In contrast, viral sequences contributed 12% and 10% to the DNA libraries of the South Atlantic and Equatorial Pacific, respectively (Supplementary Figure S2).

### Characteristics of the Sorted Viromes

Thirty-two sorted viromes were produced from seawater samples collected at 16 stations (two virus particle clusters sorted from each station) along the global deep ocean conveyor belt following the NADW and the PAABW (Figure 1). The viromes were grouped in six geographic regions according to water mass (NADW and PAABW) and location where the samples were collected (northern hemisphere, equator and southern hemisphere), resulting in six virome datasets (Supplementary Table S1). The whole genome amplification of sorted viruses yielded a total of 43 million quality-trimmed reads, assembled into 575 viral contigs (205 contigs from NADW and 370 contigs from PAABW) as predicted with VirSorter, with a minimum length of ∼1,500 bp and a maximum length of ∼18,000 bp (Table 2). Fifteen percent of the total quality-trimmed reads were mapped to the obtained viral contigs. These represented 12% of the reads from NADW and 18% of the PAABW reads (Table 2).

**TABLE 2:** Summary of the total number of contigs (N) (obtained with Virsorter, minimum length 1,500 bp), their average (avg_len), minimum (min_len) and maximum (max_len) length (bp), and%GC content of the contigs. Total number of trimmed reads (num_seqs) and their length (sum_len). Total number of mapped reads into viral contigs (num_seqs), their length (sum_len) and the% of mapped reads to the viral contigs are also indicated.

		**Contigs**					**Reads**				
		**Viral contigs mined with Virsorter**					**Trimmed Reads**		**Mapped Reads Into total viral Contigs**		
	**Samples**	**N**	**min_len (bp)**	**avg_len (bp)**	**max_len (bp)**	**GC%**	**num_seqs**	**sum_len (bp)**	**num_seqs**	**sum_len (bp)**	**% mapped reads**
Atlantic	North Atlantic	11	1,507	4,146	12,884	38	13,944,333	3,390,243,489	1,466,305	270,703,351	11
	A_Equator	48	1,673	2,793	6,407	37	5,076,934	1,134,402,129	615,029	123,249,466	12
	South Atlantic	146	1,509	3,517	18,206	36	7,319,179	1,832,146,844	983,162	199,166,371	13
Pacific	South Pacific	244	1,503	3,910	13,092	36	9,229,727	2,516,721,586	2,032,162	505,268,561	22
	P_Equator	122	1,541	3,496	8,878	36	6,073,330	1,832,829,353	1,318,215	340,742,928	22
	North Pacific	4	3,636	6,216	8,476	47	1,959,466	423,056,799	216,239	45,300,028	11

### Relative Abundance and Diversity of Viral Communities

**FIGURE 2:**
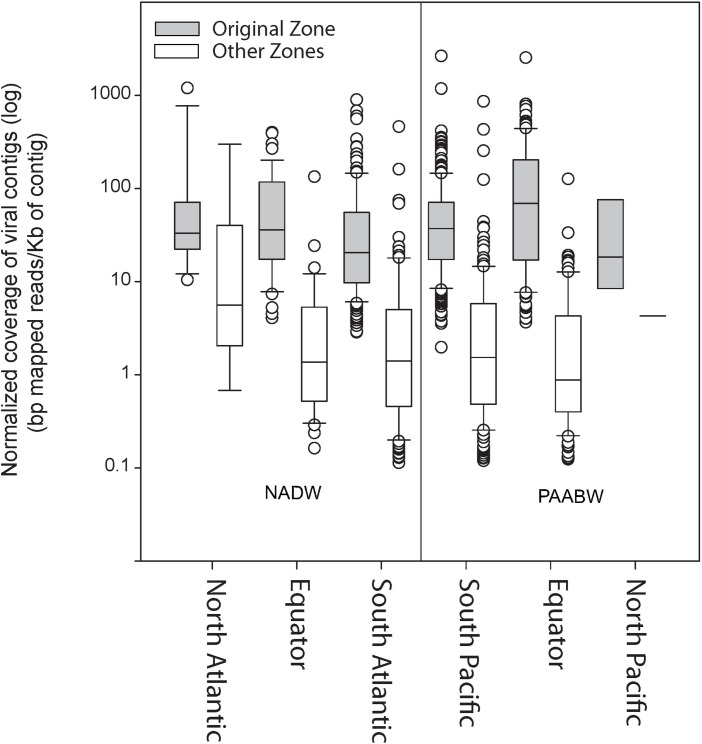
Normalized coverage of the viral contigs from each library (bp mapped per Kb of contig) at the original stations from where the sample was collected compared with their relative abundance at other stations.

Read recruitment from any of the six combined viromes to itself was higher (on average 92 bp mapped per kilobase of contigs) than the recruitment to viromes from any of the other water mass and region (11 bp mapped per kilobase of contigs) (Figure 2).

**FIGURE 3:**
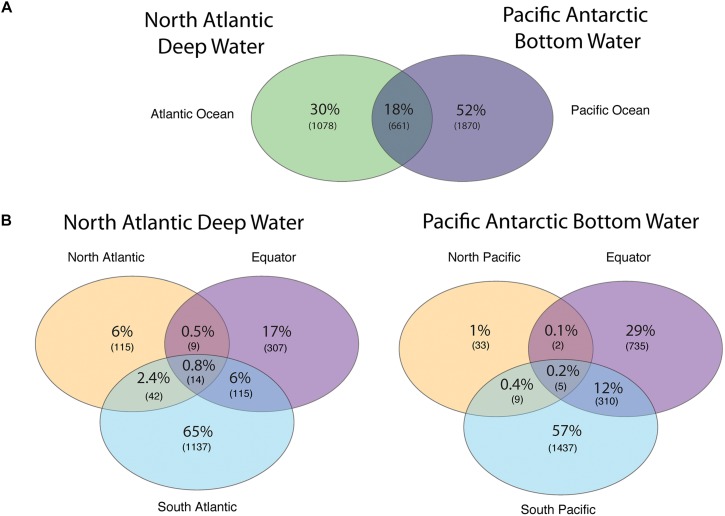
Venn diagram showing the shared and unique viral PCs obtained from subsampled reads (200,000 reads per virome) recruited into viral ORFs **(A)** between the NADW and the PAABW, and **(B)** between different regions through the thermohaline circulation of NADW and of PAABW.

The number of viral protein clusters shared between the NADW and PAABW in different oceanic regions is depicted in Figure 3. The NADW and the PAABW shared 661 protein clusters (18% of total protein clusters) (Figure 3A). PAABW harbored a larger number of unique protein clusters (1870 protein clusters, 52% of the total protein clusters) than the NADW (1078 protein clusters, 30%) (Figure 3A). NADW viromes from the North Atlantic, Equator and South Atlantic shared only 14 protein clusters (0.8% of NADW protein clusters) (Figure 3B). The South Atlantic NADW harbored the highest number of unique protein clusters, followed by the NADW at the Equator and the North Atlantic (Figure 3B). PAABW viromes shared only 5 protein clusters (0.2%) between the three regions of the Pacific Ocean (Figure 3B). Similarly, to the NADW, PAABW harbored the largest number of unique protein clusters in the South Pacific followed by the Equator and North Pacific (Figure 3B).

**FIGURE 4:**
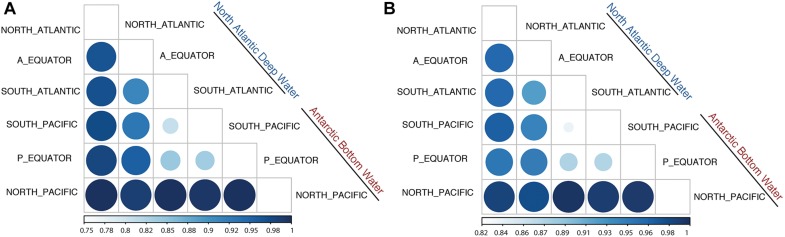
Jaccard Dissimilarity analysis of the subsampled (200,000 reads) sorted viromes **(A)** and PCs **(B)** collected in NADW and PAABW along the deep-water circulation.

Jaccard Dissimilarity analysis of the viral communities and protein clusters along the NADW and PAABW further confirmed that the viral communities differed between different regions and water masses (Figures 4A,B). Adjacent regions were generally more similar to each other than to more distant regions, i.e., a decreasing similarity can be observed between North Atlantic, the Equator and South Atlantic (Figures 4A,B) and from South Pacific northwards. The viral communities from the southern hemisphere clustered with the equatorial Pacific, while the viral communities in the northern hemisphere clustered with the equatorial Atlantic (Supplementary Figures S3A,B).

### Viral Communities Distribution Along the Deep-Water Thermohaline Circulation

**FIGURE 5:**
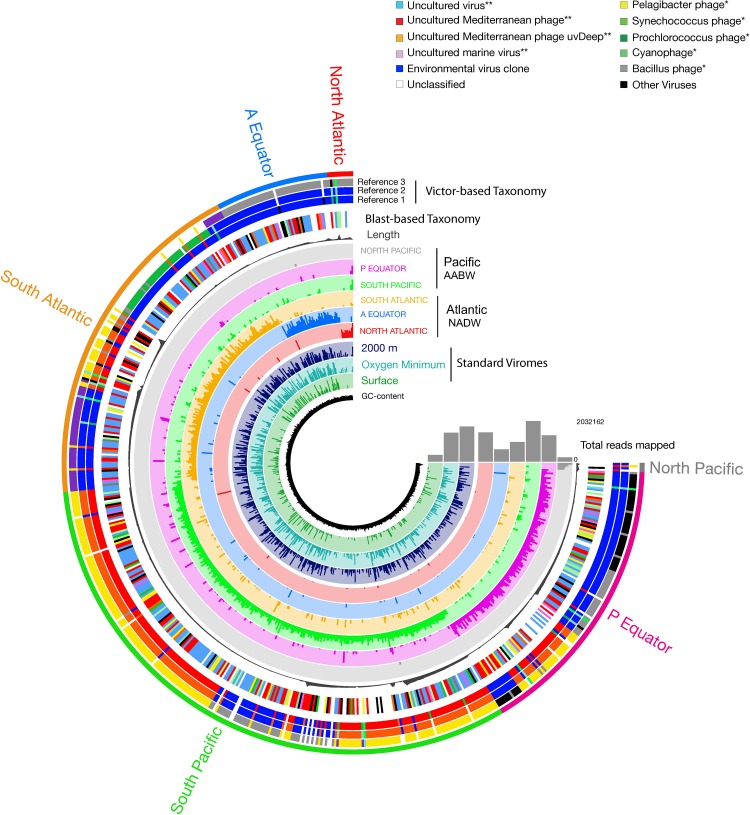
Coverage of viral contigs in NADW and PAABW samples. Coverage in the three standard virome samples from the North Atlantic surface, oxygen minimum and 2000 m is indicated in 2–4 inner rings, respectively. Length of the contigs (gray bar, in bp). The height of the colored bars within a ring represents the mean coverage (second and third quartile) of a contig in a given sample. The samples were ordered by geographic location following the deep-water conveyor belt circulation. The first outer ring indicates the taxonomic classification of the viral contigs based on BLASTx at class (^∗∗^), and at the genus (^∗^) level. The next 2 to 4 outer rings indicate the taxonomic classification based on VICTOR, showing the three most relevant identified references that clustered with the viral contigs.

Pronounced regional differences of the viral communities were detected along the deep-water circulation with most of the reads from any given virome mapping to the contigs from the same (“original”) virome (Figure 5). Some viral contigs were also ubiquitously distributed across both water masses (Figure 5 and Supplementary Table S2). Noticeably, a large fraction of the contigs obtained from the deep sorted viromes mapped to reads obtained from the surface standard virome sampled at St.0 (Figure 5). Most of the viral contigs of the NADW and PAABW were affiliated with uncultured viruses (196 contigs, 34% of the total contigs), an uncultured Mediterranean phage (123 contigs, 21%), followed by *Pelagibacter* phage (30 contigs, 5%), *Synechococcus* phage (28 contigs, 5%), uncultured marine viruses (17 contigs, 3%) and *Prochlorococcus* phage (13 contigs, 2%). Other viral groups affiliated with phages of *Bacillus, Cyanobacteria*, *Staphylococcus, Caulobacter* and *Gordonia* accounted for ∼5% of the total contigs, unassigned and other classified viruses accounted for 16 and 6% of the total viral contigs, respectively. Overall, only 22% of the viral contigs could be affiliated to cultured viruses, mainly bacteriophages. The most abundant cultured viruses found in our database were related to viruses infecting widespread hosts such *Pelagibacter, Synechococcus, and Prochlorococcus*. The VICTOR-based taxonomic classification of the viral contigs (Figure 5, Supplementary Figure S4, and Supplementary Table S3) exhibited similar trends, with the majority of the virus sequences closely related to uncultured environmental viruses (43%) and vSAGs (52%), followed by widespread cultured marine phages (3.5%) (Figure 5, Reference 1 Victor-Based Taxonomy).

A significant number of reads was tentatively affiliated with *Prochlorococcus* phages with, on average, 30 bp mapped per kilobase of contigs followed by *Synechococcus* and *Pelagicbacter phages*, with 27 and 21 bp mapped per kilobase of contigs, respectively (Figure 5 and Supplementary Figure S5). Sequences tentatively affiliated with an uncultured Mediterranean Sea phage also accounted for a significant proportion of mapped reads (69 bp mapped per kilobase of contigs, on average). Nevertheless, most of the sampled viral groups lacked cultured representatives or viral reference genomes (Figure 5 and Supplementary Figure S5).

**FIGURE 6:**
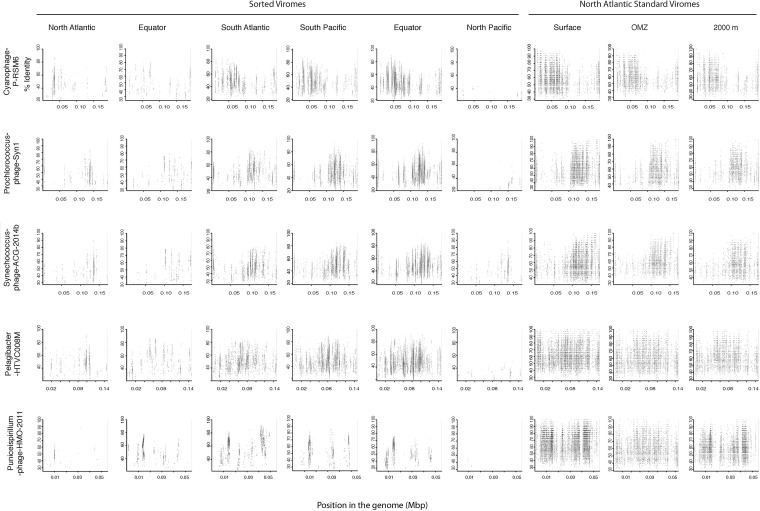
Fragment recruitment of sorted and standard viromes to selected cultured viral genomes based on tBLASTx alignments. The x-axis represents the genome size, while the y-axis represents the similarity percentage of the viral reads against the reference genome.

**FIGURE 7:**
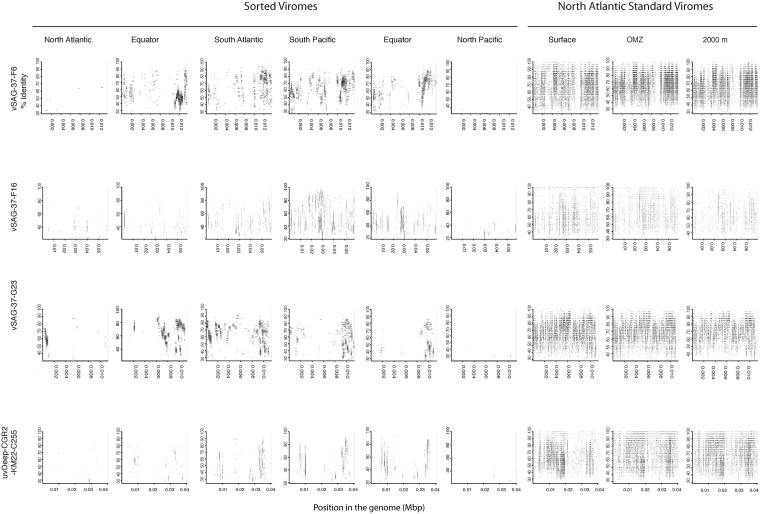
Fragment recruitment of sorted and standard viromes to selected uncultured viral genomes based on tBLASTx alignments. The x-axis represents the genome size, while the y-axis represents the similarity percentage of the viral reads against the reference genome.

Since most of the sorted virome sequences were related to specific viral genomes or environmental virus clones according to the results from the taxonomic analyses, these genomes and clones were selected for the fragment recruitment analyses of our viral reads. The recruitment analyses indicated a low coverage and percentage of similarity between the viral reads obtained in the North Atlantic, Atlantic Equator and North Pacific regions and the reference genomes of the most abundant widespread marine viruses. The larger genome coverage to cultured representatives was found in the South Atlantic, South Pacific and Pacific Equator sorted viromes and in the standard viromes (surface, OMZ and 2000 m) (Figure 6 and Supplementary Table S4). Most of the high coverage recruitments from the sorted viromes exhibited 40–80% amino acid identity, while similarity greater than 90% was only found within the standard viromes reads recruited against Pelagibacter phage HTVC008M and Puniceispirillum phage HMO-2011 (Figure 6 and Supplementary Table S4). Besides cultured representatives, viral SAGs from surface waters and uncultured marine viruses also showed a relatively high coverage to viral reads recovered from the sorted and standard viromes. The recruitment plot showed that vSAG-37-F6 and vSAG-37-G23 had the highest genome coverage within the sorted and standard viromes (Figure 7 and Supplementary Table S4), while uvDeep-CGR2-KM22-C255 showed the highest coverage and amino acid similarity in the 2000 m standard virome (Figure 7 and Supplementary Table S4).

## Discussion

### Biotic and Environmental Variables

The physico-chemical characteristics of the two water masses studied here remained constant, however, the microbial and viral abundance showed some variability, suggesting that particle flux from the photic layer or other aspects, such as *in situ* production, may locally influence the deep microbial and viral communities (Nagata et al., 2000). Additionally, the NADW, as younger water mass than the PAABW, harbored higher microbial and viral abundances than the PAABW. Thus, with aging of the water masses, microbial and viral abundance are decreasing along the meridional overturning circulation following the general concentration pattern of dissolved organic carbon (DOC) (Hansell, 2013).

Yang et al. (2014) showed that viral abundance significantly decreased from the relatively younger water mass (PAABW) to the older water mass (Pacific Deep Water), coinciding with a 0.4% of the total DOC sink in the Pacific Ocean. The pattern of the virus-to-prokaryote ratio along NADW and PAABW, together with the long turnover time of microorganisms and viruses support a DOC-like distribution of the viruses in the deep water masses. Thus, the transition from younger to older water mass might also have an effect on the viral-host interactions and virus diversity.

### Viral Diversity and Distribution Across Two Deep-Water Masses

Most of the identified viral groups were widely distributed along different regions of the thermohaline circulation, although their relative abundance was higher in the region where they were isolated (Figure 5). This finding suggests a relatively high local variability within a limited global diversity, in agreement with results from other marine viral metagenomes (Hurwitz and Sullivan, 2013; Brum et al., 2015) and a natural *Synechococcus* population (Deng et al., 2014). The low temperature of deep-sea water masses and the consequently longer turnover time of bacteria and slower viral decay in this realm may favor the dispersion of locally produced virus particles across the deep overturning circulation and support the viral seed-bank hypothesis (Breitbart and Rohwer, 2005). Under these environmental conditions, viruses may behave conservatively and thus, might reflect to some extent the different formation sites of the respective deep-water masses. Therefore, the viral composition in the PAABW might reflect the mixture of NADW, Indian Ocean Deep Water and circumpolar deep water mixing and sinking at the Antarctic shelf. In contrast, the viral composition in the NADW may initially reflect the mixture of subtropical surface waters and Arctic shelf waters (Talley, 2013) and through its way toward Antarctic waters, it probably incorporates virus from other sources through mixing with other water masses or particles sedimenting into these water masses. The overlap between surface and deep water viral communities might indicate a transport of viruses associated with sinking particles (Figure 5 and Supplementary Figure S6) across otherwise stratified waters, suggesting that the deep viral community might partially comprise surface representatives.

The widespread occurrence of some viral groups along the deep thermohaline circulation might be the result of the ubiquitous distribution of key prokaryotic phylotypes found in the deep ocean (Martin-Cuadrado et al., 2007; Sintes et al., 2013).

Archaea represent a large fraction of the microbial community in meso- and bathypelagic waters, reaching up to 30% of the prokaryotic community (Herndl et al., 2005). Recent studies have indicated that Archaea are mainly controlled by viruses in the deep seafloor, suggesting that archaeal phages play an important role in the deep-sea ecosystem (Danovaro et al., 2015, 2016; Nigro et al., 2017). Other bacterial groups such as SAR324, SAR406 also dominated the bathypelagic microbial communities (Lopez-Garcia et al., 2001; Nunoura et al., 2015). Therefore, those viruses infecting the dominant groups should be present. However, viruses infecting the main bathypelagic communities such as Archaea, SAR324 and SAR406 were not detected in NADW and PAABW.

Only a small proportion of the obtained reads were mapped to the obtained viral contigs. The scarcity of identified representatives of deep water viruses also suggests that one of the main limitations in assessing the deep ocean viral community structure is the low number of both microbial and viral genomes available from this environment (Mizuno et al., 2016).

### Sinking Particles Influence Microbial and Viral Community Composition in Deep Waters

Viral contigs associated with phages of *Synechococcus* and *Prochlorococcus* comprised a considerable fraction of viral contigs in the NADW and PAABW. Additionally, the similarity observed between deep-ocean viral reads and widespread bacteriophages infecting photosynthetic hosts (Figure 6), and the high coverage and amino acid similarity between surface and deep viral contigs (Supplementary Figure S6) support the idea of an active transport of microbes and viruses from the epipelagic realm through sinking particles as previously reported (Hurwitz et al., 2014, 2015) based on the presence of photosystem II reaction center genes (psbA) in bathypelagic viruses. In agreement with our findings, Gregory et al. (2019) suggested that the deep-sea viral populations may be more dependent on migration from sinking habitats rather than on local production.

The high virus-to-prokaryote ratio found in deep waters (Yang et al., 2014; Lara et al., 2017) also points to the transfer of viruses originated from the surface ocean to the dark ocean through sinking particles. It is unlikely that phages of cyanobacteria will find a host in the deep ocean. Hence, an ecological function of these phages is difficult to envision in that environment. However, it cannot be excluded that wide-spread viral groups and/or broad host range viruses originating from the ocean surface contribute to the mortality of deep sea microbes and, hence, affecting the carbon and nutrient cycling in the meridional overturning circulation.

It is likely that *in situ* viral production through lytic infection of abundant and active heterotrophic microbes, supported by organic sinking particles, also contribute to the high virus-to-prokaryote ratios we measured. Nevertheless, our sequencing data did not show any clear evidence concerning the life strategies of deep-sea viruses.

In aged water masses such as the North PAABW, where the available DOC is old and refractory (Hansell, 2013), sinking particles may provide the only source of organic matter available for the activity of the deep-sea microbial community (Hansell and Ducklow, 2003) and, consequently, shaping the viral communities.

## Conclusion

Sorted viromes were used to assess the changes in viral community across two major water masses NADW and PAABW of the deep thermohaline circulation. Significant differences in viral abundance and composition were found between the NADW and PAABW. The proportion of shared viral protein clusters between the two water masses together with the presence of widespread cultured and uncultured viral groups suggest that the viral distribution along the deep overturning circulation may be at least partially explained by passive dispersion. This would confirm the seed-bank hypothesis. Nevertheless, the presence of viral groups with phototrophic hosts in these bathypelagic waters also suggests that sinking particles may play an important role in shaping the deep-sea microbial community and consequently, the viral community. Therefore, sinking particles influence the virus-host interactions and the biogeochemical cycles of the deep ocean. The lack of deep-sea viral and microbial representatives remains one of the main bottlenecks in this research. It prevents the identification of the key viruses and the assessment of viral-host interactions in the dark ocean.

## Data Availability

The datasets generated for this study can be found in the DDBJ database under the accession number DRA008218.

## Author Contributions

DC, JM, and TY contributed to the concept and design of the study and wrote the manuscript. MC and YT performed the part of the bioinformatic analyses. DC analyzed the data and wrote the first draft of the manuscript. GH, ES, and TN provided feedback and contributed to the manuscript revision. DC, GH, and TN provided funding for the analyses.

## Conflict of Interest Statement

The authors declare that the research was conducted in the absence of any commercial or financial relationships that could be construed as a potential conflict of interest.

## Footnotes

1 https://jgi.doe.gov/

2 https://www.cyverse.org/

3 https://www.imicrobe.us/#/projects/334

## Supplementary Material

FIGURE S1 Representative cytograms of green fluorescence (SYBR Green I) versus side scatter and green fluorescence versus forward scatter showing the actual sorting gates (P2 and P3) employed for the two viral populations targeted in this study. Gate P5 in green fluorescence versus forward scatter was used to discriminate background within the sorting gates.

FIGURE S2 Classification of the reads obtained from the sorted viromes based on Kaiju classification with the non-redundant NCBI database.

FIGURE S3 Clustering of the sorted viromes **(A)** and PCs **(B)** based on the Jaccard Dissimilarity matrix.

FIGURE S4 Phylogenomic trees (of the sorted viromes) obtained using the Genome-BLAST Distance Phylogeny (GBDP) method, inferred using the formulas D0. The numbers above branches are the pseudo-bootstrap support values from 100 replications. The branch lengths of the resulting VICTOR trees are scaled in terms of the respective distance formula used.

FIGURE S5 Taxonomic identification and relative abundance (bp mapped per Kb of contig) of the most abundant viral contigs sampled in NADW and PAABW.

FIGURE S6 Fragment recruitment of the surface standard virome to six selected deep viral contigs (obtained in the NADW and PAABW) based on tBLASTx alignments. The x-axis represents the genome size, while the y-axis represents the similarity percentage of the viral reads against the reference genome.

TABLE S1 Physico-chemical, microbial and viral parameters sampled at different stations in the Atlantic and Pacific Ocean. All the parameters are indicated for each individual sample. The number of viruses sorted and amplified with the whole genome amplification kit (WGA) and the obtained trimmed reads are also stated. PA, prokaryotic abundance, VA, Viral abundance, VPR, Viral to prokaryotic ratio. Grouping of samples according to oceanic region: NA, North Atlantic, EA, Equatorial Atlantic, SA, South Atlantic, SP, South Pacific, EP, Equatorial Pacific, NP, North Pacific.

TABLE S2 Percentage of recruited reads against the viral contigs sampled in the NADW and AABW along the deep ocean conveyor belt.

TABLE S3 Clustering of the viral contigs against selected viral references based on the OPTSIL algorithm.

TABLE S4 Fragment recruitment results of selected viral genomes and uncultured marine phages used as references against reads obtained from the sorted and standard viromes.
