# Supplementary figures and images for: Viral Communities in the Global Deep Ocean Conveyor Belt Assessed by Targeted Viromics
Source: Front Microbiol. 2019 Aug 21;10:1801. doi: 10.3389/fmicb.2019.01801 (PMC6712177; doi:10.3389/fmicb.2019.01801)

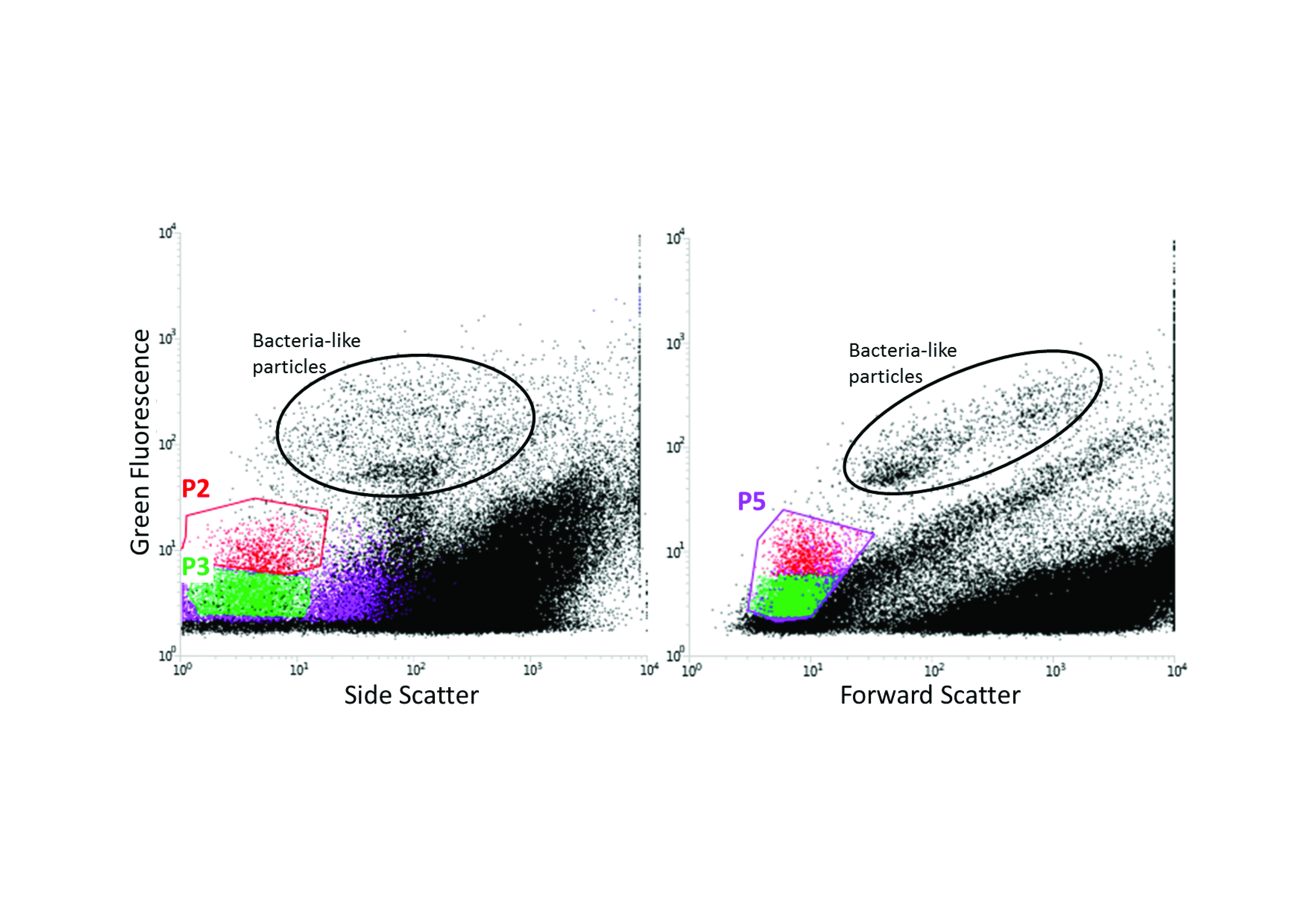

Supplement: FIGURE S1 — Representative cytograms of green fluorescence (SYBR Green I) versus side scatter and green fluorescence versus forward scatter showing the actual sorting gates (P2 and P3) employed for the two viral populations targeted in this study. Gate P5 in green fluorescence versus forward scatter was used to discriminate background within the sorting gates. [file Image_1.jpg]

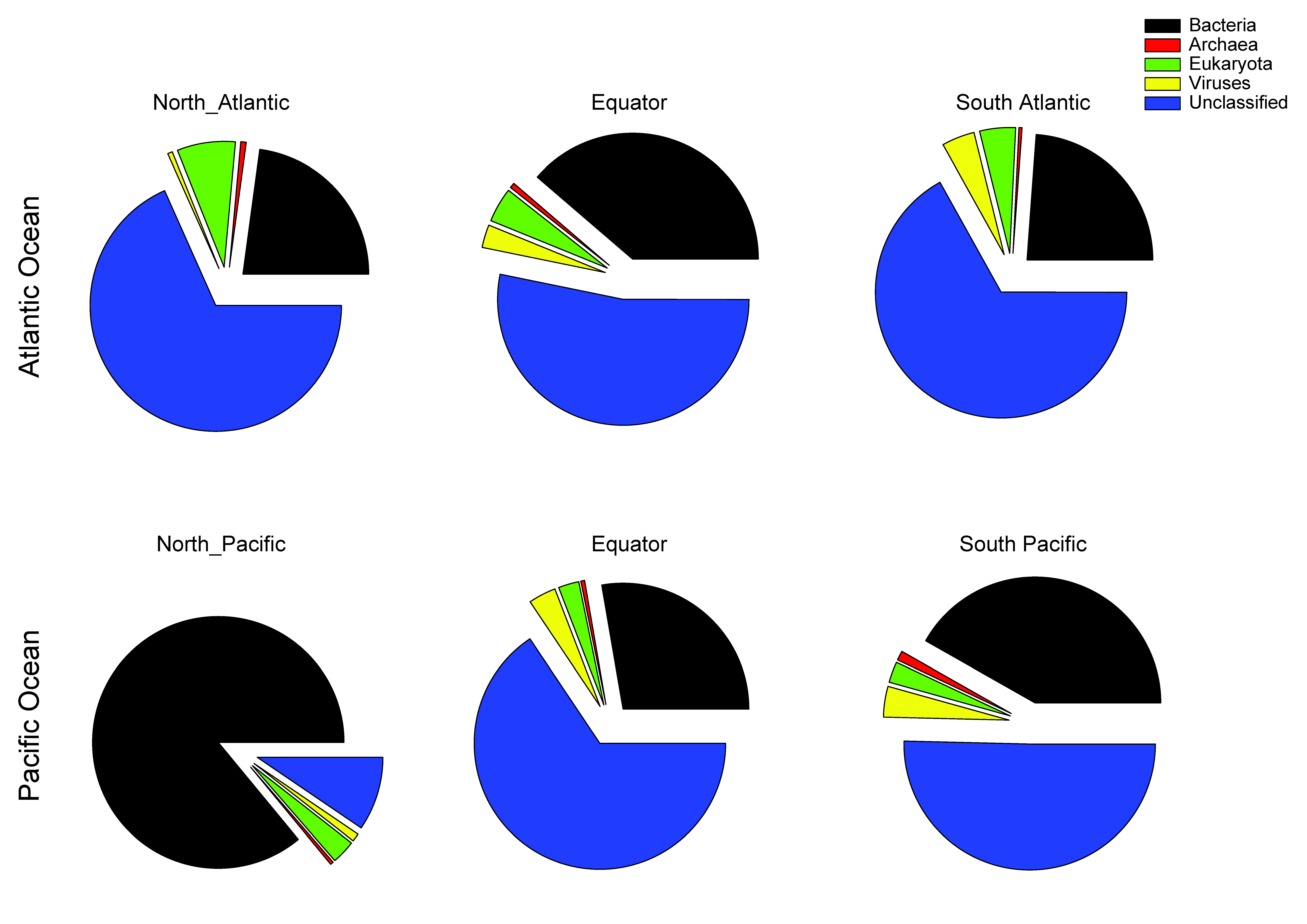

Supplement: FIGURE S2 — Classification of the reads obtained from the sorted viromes based on Kaiju classification with the non-redundant NCBI database. [file Image_2.jpg]

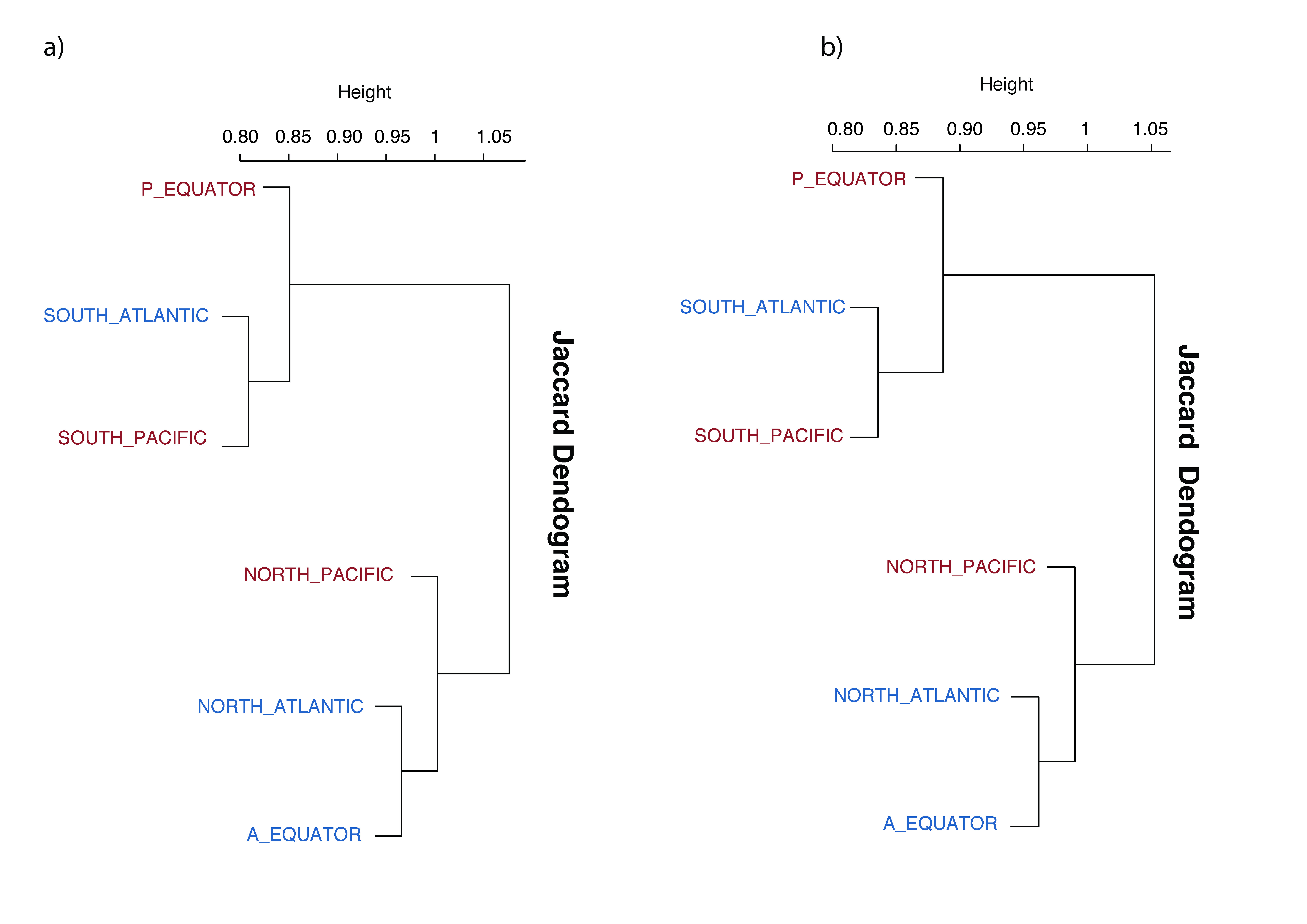

Supplement: FIGURE S3 — Clustering of the sorted viromes (A) and PCs (B) based on the Jaccard Dissimilarity matrix. [file Image_3.jpg]

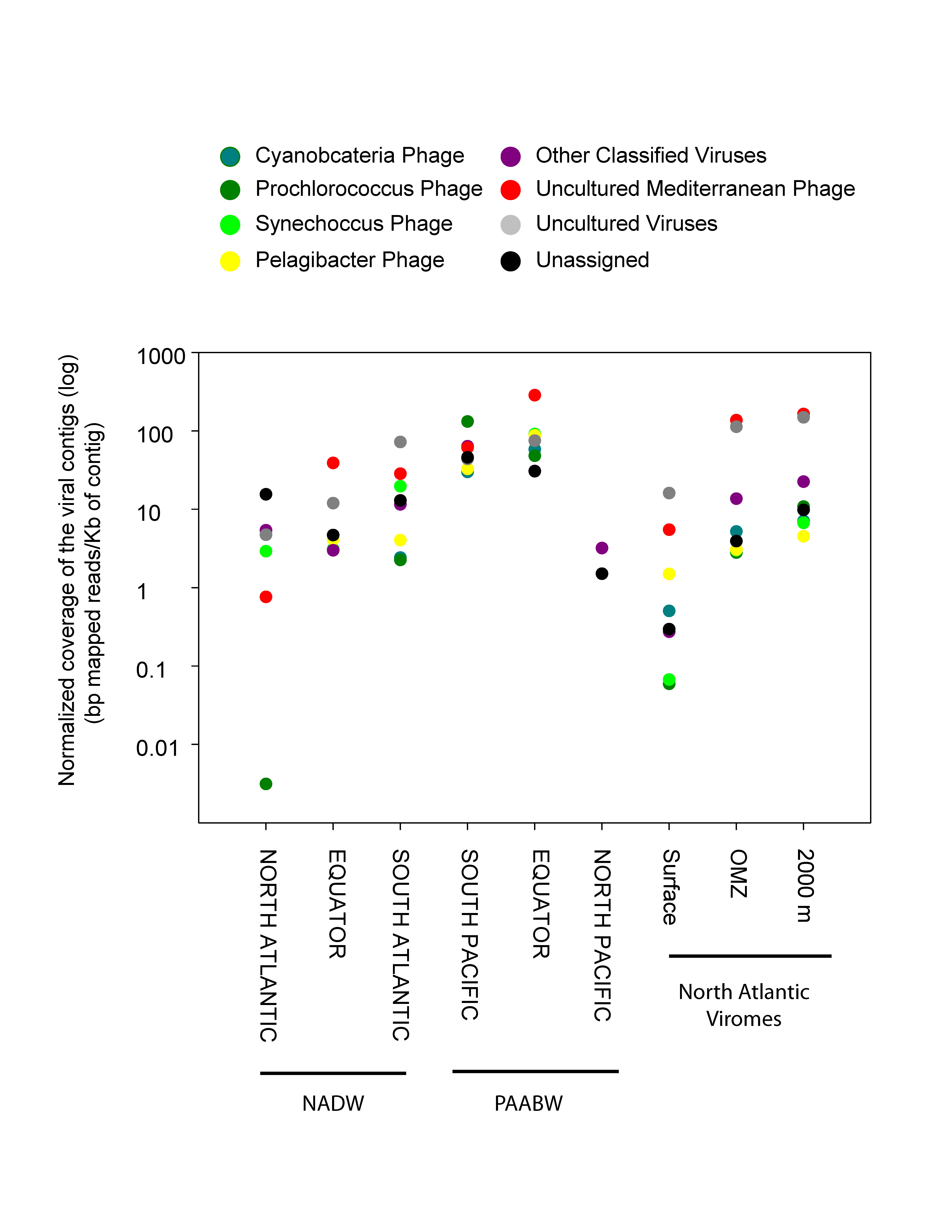

Supplement: FIGURE S5 — Taxonomic identification and relative abundance (bp mapped per Kb of contig) of the most abundant viral contigs sampled in NADW and PAABW. [file Image_5.jpg]

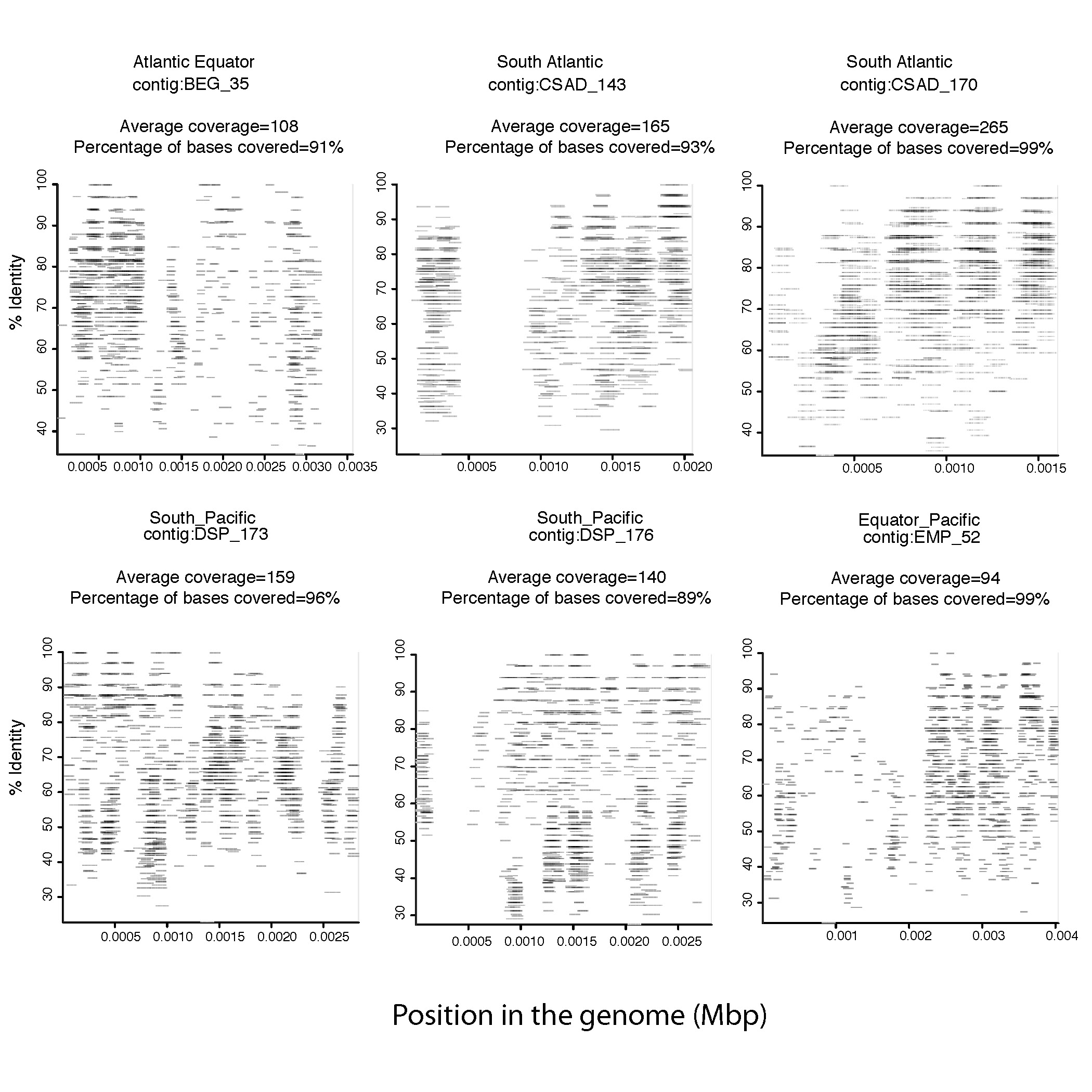

Supplement: FIGURE S6 — Fragment recruitment of the surface standard virome to six selected deep viral contigs (obtained in the NADW and PAABW) based on tBLASTx alignments. The x-axis represents the genome size, while the y-axis represents the similarity percentage of the viral reads against the reference genome. [file Image_6.jpg]
