# Supplementary material for: Viral Communities in the Global Deep Ocean Conveyor Belt Assessed by Targeted Viromics
Source: Front Microbiol. 2019 Aug 21;10:1801. doi: 10.3389/fmicb.2019.01801 (PMC6712177; doi:10.3389/fmicb.2019.01801)
Supplement: TABLE S1 — Physico-chemical, microbial and viral parameters sampled at different stations in the Atlantic and Pacific Ocean. All the parameters are indicated for each individual sample. The number of viruses sorted and amplified with the whole genome amplification kit (WGA) and the obtained trimmed reads are also stated. PA, prokaryotic abundance, VA, Viral abundance, VPR, Viral to prokaryotic ratio. Grouping of samples according to oceanic region: NA, North Atlantic, EA, Equatorial Atlantic, SA, South Atlantic, SP, South Pacific, EP, Equatorial Pacific, NP, North Pacific. [file Table_1.docx]

| Sample_ID | Population | | Station | Region | Latitude | Longitude | Viral Particles  WGA amplified | Depth  (m) | Temperature  ºC | Salinity | Oxygen  µmol/kg | PA  mL ^-1^ | VA  mL^-1^ | VPR | Trimmed Reads |
| --- | --- | --- | --- | --- | --- | --- | --- | --- | --- | --- | --- | --- | --- | --- | --- |
| 1 | P2 | St.1 | | NA | 39.49661 | -48.08546 | 942 | 2495 | 3.4240 | 34.9490 | 238.2 | 11365 | 611000 | 54 | 1744762 |
| 1 | P3 | | St.1 |  | 39.49661 | -48.08546 | 5000 | 2495 | 3.4240 | 34.9490 | 238.2 | 11365 | 611000 | 54 | 3450772 |
| 2 | P2 | | St.2 |  | 29.61556 | -66.52952 | 5000 | 2497 | 3.0880 | 34.9550 | 235.9 | 5516 | 352000 | 64 | 2668717 |
| 2 | P3 | | St.2 |  | 29.61556 | -66.52952 | 5000 | 2497 | 3.0880 | 34.9550 | 235.9 | 5516 | 352000 | 64 | 1148164 |
| 3 | P2 | | St.3 |  | 21.77658 | -61.84356 | 2312 | 2501 | 3.0450 | 34.9550 | 231.5 | 7621 | 506000 | 66 | 1011864 |
| 3 | P3 | | St.3 |  | 21.77658 | -61.84356 | 5000 | 2501 | 3.0450 | 34.9550 | 231.5 | 7621 | 506000 | 66 | 1425457 |
| 4 | P2 | | St.4 |  | 14.88004 | -54.803 | 5000 | 2502 | 3.0190 | 34.9510 | 231.0 | 7451 | 449000 | 60 | 1224963 |
| 4 | P3 | | St.4 |  | 14.88004 | -54.803 | 4553 | 2502 | 3.0190 | 34.9510 | 231.0 | 7451 | 449000 | 60 | 1269634 |
| 5 | P2 | | St.5 | EA | 3.97464 | -43.75268 | 3844 | 2756 | 2.8910 | 34.9410 | 230.6 | 9795 | 445000 | 45 | 1552155 |
| 5 | P3 | | St.5 |  | 3.97464 | -43.75268 | 5000 | 2756 | 2.8910 | 34.9410 | 230.6 | 9795 | 445000 | 45 | 1237145 |
| 6 | P2 | | St.6 |  | -0.1915 | -32.8745 | 1140 | 2529 | 3.0184 | 34.9407 | 249.4 | 20992 | 266000 | 13 | 1131180 |
| 6 | P3 | | St.6 |  | -0.1915 | -32.8745 | 3808 | 2529 | 3.0184 | 34.9407 | 249.4 | 20992 | 266000 | 13 | 1156454 |
| 7 | P2 | | St.7 | SA | -9.1608 | -28.0012 | 1148 | 2529 | 2.9670 | 34.9260 | 240.9 | 12102 | 450000 | 37 | 1313678 |
| 7 | P3 | | St.7 |  | -9.1608 | -28.0012 | 3531 | 2529 | 2.9670 | 34.9260 | 240.9 | 12102 | 450000 | 37 | 2017967 |
| 8 | P2 | | St.8 |  | -32.092 | -37.4563 | 5000 | 2534 | 3.0264 | 34.9166 | 241.4 | 18303 | 735000 | 40 | 501133 |
| 8 | P3 | | St.8 |  | -32.092 | -37.4563 | 5000 | 2534 | 3.0264 | 34.9166 | 241.4 | 18303 | 735000 | 40 | 474306 |
| 9 | P2 | | St.9 |  | -39.9648 | -42.4232 | 2443 | 3047 | 1.6703 | 34.7519 | 201.2 | 35359 | 921000 | 26 | 2000294 |
| 9 | P3 | | St.9 |  | -39.9648 | -42.4232 | 5000 | 3047 | 1.6703 | 34.7519 | 201.2 | 35359 | 921000 | 26 | 1011801 |
| 10 | P2 | | St.10 | SP | -40.0125 | -169.9955 | 5000 | 3999 | 1.0523 | 34.7123 | 195.8 | 9020 | 115100 | 13 | 885378 |
| 10 | P3 | | St.10 |  | -40.0125 | -169.9955 | 1943 | 3999 | 1.0523 | 34.7123 | 195.8 | 9020 | 115100 | 13 | 572528 |
| 11 | P2 | | St.11 |  | -34.996 | -170.0036 | 5000 | 3999 | 1.3259 | 34.7183 | 189.5 | 7269 | 125800 | 17 | 753708 |
| 11 | P3 | | St.11 |  | -34.996 | -170.0036 | 5000 | 3999 | 1.3259 | 34.7183 | 189.5 | 7269 | 125800 | 17 | 885715 |
| 12 | P2 | | St.12 |  | -30.0015 | -169.9988 | 5000 | 4001 | 1.2119 | 34.716 | 191.6 | 8609 | 155400 | 18 | 1031278 |
| 12 | P3 | | St.12 |  | -30.0015 | -169.9988 | 5000 | 4001 | 1.2119 | 34.716 | 191.6 | 8609 | 155400 | 18 | 1016449 |
| 13 | P2 | | St.13 |  | -19.9995 | -170.0011 | 5000 | 3999 | 1.3241 | 34.7149 | 187.3 | 7322 | 134400 | 18 | 2150029 |
| 13 | P3 | | St.13 |  | -19.9995 | -170.0011 | 5000 | 3999 | 1.3241 | 34.7149 | 187.3 | 7322 | 134400 | 18 | 1934638 |
| 14 | P2 | | St.14 | EP | 0.00333 | -169.9983 | 5000 | 4000 | 1.3949 | 34.6931 | 148.2 | 6879 | 120400 | 18 | 679139 |
| 14 | P3 | | St.14 |  | 0.00333 | -169.9983 | 5000 | 4000 | 1.3949 | 34.6931 | 148.2 | 6879 | 120400 | 18 | 822455 |
| 15 | P2 | | St.15 |  | 5.08883 | -170.0075 | 5000 | 4000 | 1.4084 | 34.6916 | 143.9 | 8746 | 137400 | 16 | 2498069 |
| 15 | P3 | | St.15 |  | 5.08883 | -170.0075 | 5000 | 4000 | 1.4084 | 34.6916 | 143.9 | 8746 | 137400 | 16 | 2076667 |
| 16 | P2 | | St.16 | NP | 10.00 | -170.0011 | 5000 | 4000 | 1.4445 | 34.6852 | 150.8 | 5138 | 78800 | 15 | 1075930 |
| 16 | P3 | | St.16 |  | 10.00 | -170.0011 | 5000 | 4000 | 1.4445 | 34.6852 | 150.8 | 5138 | 78800 | 15 | 883536 |

**Table S1**. Physico-chemical, microbial and viral parameters sampled at different stations in the Atlantic and Pacific Ocean. All the parameters are indicated for each individual sample. The number of viruses sorted and amplified with the whole genome amplification kit (WGA) and the obtained trimmed reads are also stated. PA-prokaryotic abundance, VA-Viral abundance, VPR-Viral to prokaryotic ratio. Grouping of samples according to oceanic region: NA: North Atlantic, EA: Equatorial Atlantic, SA: South Atlantic, SP: South Pacific, EP: Equatorial Pacific, NP: North Pacific.
