# Supplementary material for: Viral Communities in the Global Deep Ocean Conveyor Belt Assessed by Targeted Viromics
Source: Front Microbiol. 2019 Aug 21;10:1801. doi: 10.3389/fmicb.2019.01801 (PMC6712177; doi:10.3389/fmicb.2019.01801)
Supplement: TABLE S2 — Percentage of recruited reads against the viral contigs sampled in the NADW and AABW along the deep ocean conveyor belt. [file Table_2.docx]

**Table S2.** Percentage of recruited reads against the viral contigs sampled in the NADW and AABW along the deep ocean conveyor belt.

|  |  |  | Viral Contigs | | | | | |
| --- | --- | --- | --- | --- | --- | --- | --- | --- |
|  |  |  | North Atlantic | A_Equator | South Atlantic | South Pacific | P_Equator | North Pacific |
| Reads | Targeted Viromics | North Atlantic | 23.86 | 2.68 | 15.01 | 33.53 | 4.18 | 0.00 |
|  |  | A_Equator | 8.45 | 59.30 | 16.42 | 12.80 | 3.02 | 0.00 |
|  |  | South Atlantic | 7.53 | 0.75 | 75.49 | 13.80 | 2.42 | 0.00 |
|  |  | South Pacific | 2.65 | 0.73 | 6.72 | 85.47 | 3.00 | 0.02 |
|  |  | P_Equator | 2.01 | 0.24 | 8.83 | 10.99 | 77.52 | 0.00 |
|  |  | North Pacific | 16.75 | 0.15 | 16.33 | 50.33 | 0.17 | 16.26 |
|  | Viromes | Surface | 0.26 | 21.47 | 32.48 | 35.45 | 10.34 | 0.00 |
|  |  | Oxygen Minimum | 0.14 | 13.28 | 37.26 | 37.86 | 11.40 | 0.00 |
|  |  | 2000 m | 0.08 | 11.00 | 33.76 | 41.66 | 13.13 | 0.00 |
