# Supplementary material for: Viral Communities in the Global Deep Ocean Conveyor Belt Assessed by Targeted Viromics
Source: Front Microbiol. 2019 Aug 21;10:1801. doi: 10.3389/fmicb.2019.01801 (PMC6712177; doi:10.3389/fmicb.2019.01801)
Supplement: TABLE S4 — Fragment recruitment results of selected viral genomes and uncultured marine phages used as references against reads obtained from the sorted and standard viromes. [file Table_4.docx]

|  |  |  | **Sorted Viromes** | | | | | | | | | | |  | **Standard Viromes** | | | | | |
| --- | --- | --- | --- | --- | --- | --- | --- | --- | --- | --- | --- | --- | --- | --- | --- | --- | --- | --- | --- | --- |
|  |  |  | **North Atlantic** | | **Equator** | | **South Atlantic** | | **South Pacific** | | **Equator** | | **North Pacific** | | **Surface** | | **OMZ** | | **2000 m** | |
| Genome Name | NCBI ID | Length (bp) | Avg. Cov. | %Cov. | Avg. Cov. | %Cov. | Avg. Cov. | %Cov. | Avg. Cov. | %Cov. | Avg. Cov. | %Cov. | Avg. Cov. | %Cov. | Avg. Cov. | %Cov. | Avg. Cov. | %Cov. | Avg. Cov. | %Cov. |
| Cyanophage P-RSM6 | NC_020855.1 | 192497 | 2.91 | 8.75 | 0.93 | 7.88 | 5.15 | 23.45 | 8.79 | 25.41 | 29.08 | 24.51 | 0.09 | 1.31 | 4.99 | 32.11 | 2.74 | 27.64 | 2.04 | 23.99 |
| Prochlorococcus phage Syn1 | NC_015288.1 | 191195 | 2.93 | 8.85 | 0.40 | 6.68 | 5.88 | 21.30 | 9.59 | 24.02 | 27.83 | 23.35 | 0.25 | 1.69 | 4.90 | 31.44 | 2.38 | 24.83 | 1.62 | 21.36 |
| Synechococcus phage ACG-2014b | NC_027130.1 | 172688 | 2.97 | 9.63 | 0.52 | 7.44 | 5.00 | 24.27 | 9.40 | 28.54 | 29.31 | 27.64 | 0.20 | 2.08 | 5.82 | 39.29 | 2.77 | 30.46 | 1.86 | 26.04 |
| Pelagibacter phage HTVC008M | NC_020484.1 | 147284 | 3.79 | 15.22 | 1.54 | 14.74 | 7.12 | 32.68 | 13.68 | 48.10 | 42.79 | 47.70 | 0.52 | 4.25 | 10.25 | 66.45 | 6.23 | 58.76 | 4.49 | 49.86 |
| Puniceispirillum phage HMO-2011 | NC_021864.1 | 55282 | 0.34 | 4.67 | 4.37 | 14.81 | 8.29 | 42.26 | 4.46 | 24.10 | 9.80 | 19.27 | 0.02 | 0.94 | 43.17 | 78.12 | 31.67 | 74.70 | 22.07 | 66.28 |
| vSAG-37-F6 | KY052810.1 | 13589 | 0.09 | 8.49 | 18.48 | 59.73 | 9.89 | 63.36 | 23.70 | 70.78 | 19.15 | 58.78 | 0.04 | 3.11 | 110.07 | 96.86 | 241.57 | 97.46 | 210.40 | 94.74 |
| vSAG-37-F16 | KY052811.1 | 58722 | 0.59 | 8.73 | 7.90 | 12.34 | 5.50 | 30.74 | 19.18 | 57.12 | 16.72 | 30.75 | 3.01 | 6.46 | 8.1 | 61.13 | 8.98 | 68.53 | 5.42 | 51.11 |
| vSAG-37-G23 | KY052812.1 | 11379 | 10.43 | 20.01 | 24.69 | 46.93 | 25.97 | 84.69 | 12.26 | 63.85 | 10.59 | 32.48 | 0.16 | 5.18 | 123.09 | 97.57 | 118.18 | 99.07 | 70.14 | 96.84 |
| uvDeep-CGR2-KM22-C255 | KT997865.1 | 40440 | 0.57 | 4.65 | 1.38 | 13.23 | 6.00 | 25.38 | 6.73 | 18.42 | 10.05 | 23.89 | 0.09 | 3.77 | 31.97 | 79.97 | 35.11 | 94.88 | 38.06 | 94.34 |

**Table S4.** Fragment recruitment results of selected viral genomes and uncultured marine phages used as references against reads obtained from the sorted and standard viromes.
